# Vincent Middleton Coates

**Published:** 1934

**Authors:** 


### Obituary

### VINCENT MIDDLETON COATES, M.C., M.D., M.R.C.P.

THE tragic death of Dr. Vincent Coates has deprived the West Country of a well-known and distinguished figure. Although he himself was born in Edinburgh, all his family associations were with the West Country. His grandfather, Dr. C. M. Coates, was a well-known physician in Bath, while his father, Dr. C. Middleton Coates, spent all his professional life in Somerset. Vincent Coates himself had carried on a successful practice as a physician in his grandfather's house in Bath since 1919.

Coates first sprang to fame as a Rugby footballer. Educated at Monkton Combe and Haileybury, he got his colours during his first year and later captained the latter school. In 1907 he went into residence at Gonville and Caius College, Cambridge, and won his Rugger Blue as a freshman. Knee trouble kept him out of the Varsity team the following year, but he played several times in 1909 although still somewhat hampered, and was a tower of strength for the College as left wing three-quarter both in 1909 and 1910. He played also many times for Bath and for Somerset, and in 1913 was awarded his international cap, playing for England in all five matches.

Coates entered the Bristol Medical School in 1912, working at the Royal Infirmary. He qualified in Medicine in 1915 and immediately joined the R.A.M.C. In 1916 he was awarded the Military Cross in France, and subsequently went to Salonika, where he was in charge of a travelling bacteriological unit. After demobilization he worked for a time at the Royal

Infirmary, Bristol, and was appointed Physician to the Bristol Hospital for Women and Children and Clinical Lecturer in Medicine in the University of Bristol. Shortly after settling in Bath he was appointed Physician to the Royal Mineral Water Hospital and Assistant Physician to the Royal United Hospital, and last April became full Physician to the latter institution. He was Consulting Physician to the Trowbridge and Freshford Hospitals.

Coates, who was an M.D. of Cambridge and a member of the College of Physicians, will be remembered as a brilliant physician. His chief interest was in the rheumatic group of diseases: he had contributed many important papers to medical literature on this subject, and with J. Delicati had published a book on rheumatoid arthritis and its treatment. He took a prominent part in all movements for the promotion of the study of rheumatic diseases and their treatment both in this country and abroad, being a member of the committee of the Royal College of Physicians on arthritis. He was Secretary to the section of Diseases of Children at the British Medical Association Meeting in Bath in 1925. His many friends and colleagues will mourn his loss and miss the stimulating contact with his original and energetic mind.

In 1915 Coates married Ethel Bertha Longuet Layton, a Bristol Royal Infirmary sister, to whom our deepest sympathy is extended.

